# Early postoperative evaluation of an open-source digital workflow for designing custom-made zirconia membranes in maxillary guided bone regeneration

**DOI:** 10.1186/s12903-025-06592-0

**Published:** 2025-07-18

**Authors:** Muhammad Ibrahim Sakr, Ahmed Sobhy Salem, Ayman Ahmed Yaseen, Mohamed Abdel-Monem Tawfik, Noha Ahmed Mansour

**Affiliations:** 1 Oral and Maxillofacial Surgery Department, Faculty of Dentistry, Mansoura University, Mansoura, Egypt; 2 Oral and Maxillofacial Surgery Department, Faculty of Oral and Dental Medicine, Delta University for Science and Technology, International costal road, Gamasa, Dakahlia Governorate, Egypt

**Keywords:** Guided bone regeneration, Zirconia, Open-source software, Bone atrophy, Maxillary ridge augmentation

## Abstract

**Background:**

Computer-guided surgery has played a crucial role in planning alveolar ridge augmentation. In the last decade, various software programs have been used in computer-guided fabricated nonresorbable membranes, including zirconia membranes, for guided bone regeneration. However, most of these software programs are not free of charge.

**Objectives:**

This study aimed to evaluate clinically and radiographically the accuracy of an open-source digital workflow for designing custom-made zirconia membranes for maxillary guided bone regeneration.

**Materials and methods:**

Twelve custom-made zirconia membranes were designed for 12 patients with maxillary alveolar defects via the integration of two free open-source software programs (Blue Sky Plan^®^ and Autodesk Meshmixer^®^) via a preoperative cone beam computed tomography scan. All patients underwent maxillary alveolar bone augmentation via the designed membranes and particulate mixtures of 1:1 autogenous and xenogenic bone grafts. The membranes were evaluated intraoperatively and radiographically via an immediate postoperative cone beam computed tomography scan, and the collected data were statistically analysed.

**Results:**

All the membranes had accurate intraoperative fits, and there were no significant differences between the virtual and milled (actual) membranes in either the volumetric analysis or the linear horizontal and vertical measurements; the P values were (0.628, 0.226 and 0.239), respectively. The designing time was significantly reduced from 4 h for the first case to 22 min for the final case, while the (mean ± standard deviation) milling time was 28 min ± 11 min, and the (mean ± standard deviation) time of the whole digital workflow including membrane sterilization was 5 h and 20 min ± 1 h and 15 min.

**Conclusion:**

Zirconia membranes can be designed with free open-source software with outstanding clinical fit and promising radiographic results. Further research should be performed with larger sample sizes and other rigid Guided Bone Regeneration membrane materials.

**Trial registration number:**

NCT06227455.

**Trial first posted date:**

26/1/2024 (Retrospectively registered).

**Supplementary Information:**

The online version contains supplementary material available at 10.1186/s12903-025-06592-0.

## Background

Guided Bone Regeneration (GBR) has emerged as a firmly established method for alveolar defect reconstruction. Its biological principle involves mechanically preventing soft tissues from infiltrating an osseous defect via the tenting effect, thereby allowing only osteoblasts and other bone cells to populate the defect [1]. This action encourages bone growth and improves the results of the bone grafting process [2]. 

Nonresorbable barriers such as extended polytetrafluoroethylene (e-PTFE), high-density PTFE (d-PTFE), titanium-reinforced e-PTFE, and titanium meshes (ready-made, prefabricated, and custom-made either by computer-aided design\computer-aided manufacturing (CAD\CAM) or 3D printing) have been reported as reliable barriers at large defects owing to their outstanding mechanical properties, which prevent soft tissue collapse and ensure defect regeneration [3]. Despite the aforementioned results, high wound dehiscence rates have been reported, which may be attributed to the sharp edges and corners caused by presurgical shaping, bending, and trimming. In addition, the excessive cost of most of these membranes hinders their widespread use [2–5]. 

The high-resolution imaging capabilities of Cone Beam Computed Tomography (CBCT) provide accurate evaluation of the alveolar bone dimensions and morphology, which is crucial for optimal treatment planning in implant dentistry [6, 7]. Compared with traditional imaging methods, CBCT offers reduced radiation exposure, abbreviated scanning duration, and improved accessibility, establishing it as the favoured option for clinicians. This imaging technique enables the production of precise 3D models essential for the design and fabrication of surgical guides via Computer-Aided Design and Manufacturing (CAD/CAM) technology [6, 8, 9]. 

Ceramic materials have recently been used as inert, inorganic, and high-strength spaces to maintain barriers in GBR [10]. One of these materials is zirconia, which is used in prosthetic fixed partial and complete dentures and dental implants. In addition to its high mechanical properties, as it has high flexural strength even at very thin thicknesses, it provides superior induction of the fibroblastic response, less biofilm adhesion, and a weaker inflammatory response than does titanium mesh [11]. The use of 3D-customized zirconia barriers in GBR protocols may be of immense value in overcoming the limitations of nonresorbable barriers and titanium meshes in alveolar ridge augmentation [12, 13]. 

Many studies have introduced the use of customized patient membranes. Most of these studies have used variable dedicated CAD software for implant planning, model segmentation, and the design of patient customized membranes, such as 3D-printed titanium meshes, milled zirconia membranes or poly-ether-ether-ketone (PEEK) [13–15]. Other studies have revealed the use of these software programs to segment and print 3D models of patients’ own jaws that can be used to prebend and prepare ready-made membranes that are either resorbable or nonresorbable before placement at the site of the defect [15, 16]. Despite the excellent features and results of these software programs, many have limited availability because of the excessive cost needed. In addition, all these studies have evaluated the accuracy of the membranes at the final stages of bone formation on the basis of the amount of bone volume [10, 12, 13]. 

This study aimed to develop and evaluate an open-source digital workflow for designing and fabrication of custom-made zirconia membranes used in maxillary GBR. In particular, the study sought to assess the immediate postoperative clinical and radiographic accuracy of the membrane design using two integrated open-source software tools.

## Methodology

### Sample size calculation

The sample size calculation was based on a dimensional accuracy evaluation of customized bone augmentation combined with 3D-printed individualized titanium mesh retrieved from previous research by Li et al. [14] The G power program version 3.1.9.7 was used to calculate the sample size on the basis of an effect size of 0.903 on the basis of the expected difference between pre- and postintervention, which was 123.4 ± 136.62. Using a 2-tailed test, α error = 0.05 and power = 80%, the total calculated sample size was at least 12.

### Patient selection and eligibility criteria

Participants were recruited through social network advertisements and posters displayed in an outpatient clinic at Faculty of Dentistry, Mansoura University. The research procedure was thoroughly explained to the participants, who were provided with written information about the study and signed a written informed consent form. This clinical study was approved by the Dental Research Ethics Committee at the Faculty of Dentistry, Mansoura University (approval number No. A02030123). The study was retrospectively registered in the clinical trial registration database (www.clinicaltrials.gov) under identification number (NCT06227455). The study commenced on (February 1, 2023) and was officially completed on (August 1, 2024). Exclusion criteria including patients complaining from any systematic disease that might complicate the surgical procedure, smokers, and pregnant females. After two months of advertising, 18 patients were recruited for the study, following inclusion and exclusion criteria, twelve patients (6 males and 6 females) seeking maxillary alveolar ridge rehabilitation were recruited for the study.

### Study variables

- The predictor value is the integration of two free open-source software programs (Blue Sky Plan^®^ and Autodesk Meshmixer^®^) to design a GBR zirconia membrane.
- The outcome variable is the accuracy of the milled designed membrane.

### Inclusion criteria

Patients with single or multiple missing maxillary teeth with vertical alveolar defects (≤ 4 mm), severe horizontal defects (≤ 6 mm), and/or both types of defects. Additionally, the study included patients who experienced a clinically apparent increase in the interarch space relative to the adjacent teeth, which could prevent the placement of any implant or prosthetic appliance in the maxilla. Patients were free of any relevant systemic disease that might contraindicate surgical intervention, and patients were free of periodontal disease, infection or any pathological lesion in the area of interest.

### Preoperative preparation

A brief dental and medical history followed by a thorough clinical examination were conducted for all patients to ensure that every patient was primarily fit for the study inclusion criteria. Periapical radiographs were used as a primary radiographic investigation to exclude the presence of any lesion in the area of interest. Finally, a CBCTscan was performed to assess the alveolar ridge volume. All CBCT scans were performed via a Veraview^®^ X800 CBCT machine (J. MORITA MFG. CORP., Tokyo, Japan). Imaging was conducted with a field of view of Ø 80 x H 100 mm, a voxel size of (160 × 160 × 160 μm), and a resolution of 2.5 Line Pairs per Millimeter (LP/mm) at 10% Modulation Transfer function (MTF). The exposure parameters included a tube voltage of 90 Kilo-volt (kV), a tube current of 8 Milliampere (mA), and a 360° rotation with an exposure time of 17.9 seconds.

### Digital workflow of membrane design

**Fig. 1:**
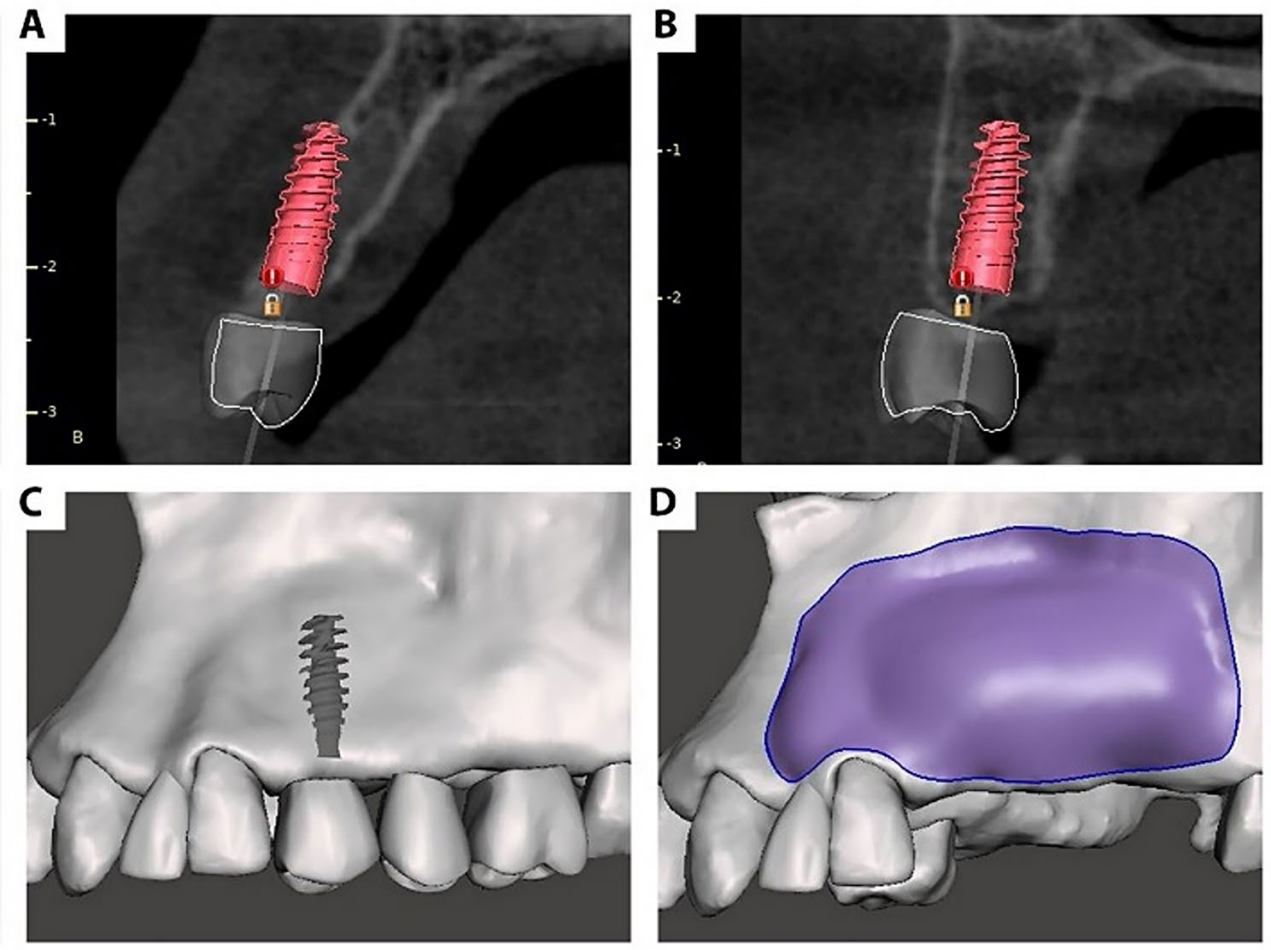
(**a**) & (**b**) Screenshots from Blue Sky Plan revealing a cross-sectional view of the alveolar defect with the virtual prosthesis and implant in place, (**c**) a screenshot from Autodesk Meshmixer showing the exported preoperative STL model of the maxilla, virtual implants, and virtual prosthesis, and (**d**) the virtually augmented model (highlighted area represents the virtually augmented area)

Several integrative steps between two specialized software programs were included as follows:

First, Digital Imaging and Communications in Medicine (DICOM) files were imported into the first open-source software, Blue Sky Plan^®^ (Blue Sky Bio, Illinois, US). After preoperative radiographic assessment, the (Advanced) module was selected, automatic jaw segmentation was performed to produce a maxillary 3D model, and virtual tooth/teeth wax up in the ideal prosthetic position was performed to perform prosthetic-driven implant planning. The desired implant(s) size was selected and virtually positioned in the proper position. In the advanced mode, automatic jaw segmentation was performed to produce a maxillary 3D model. The maxillary bony model, implant, and crown(s) were then exported as high-quality standard tessellation language (STL) files. (Figure 1a and b, and 1c)

**Fig. 2:**
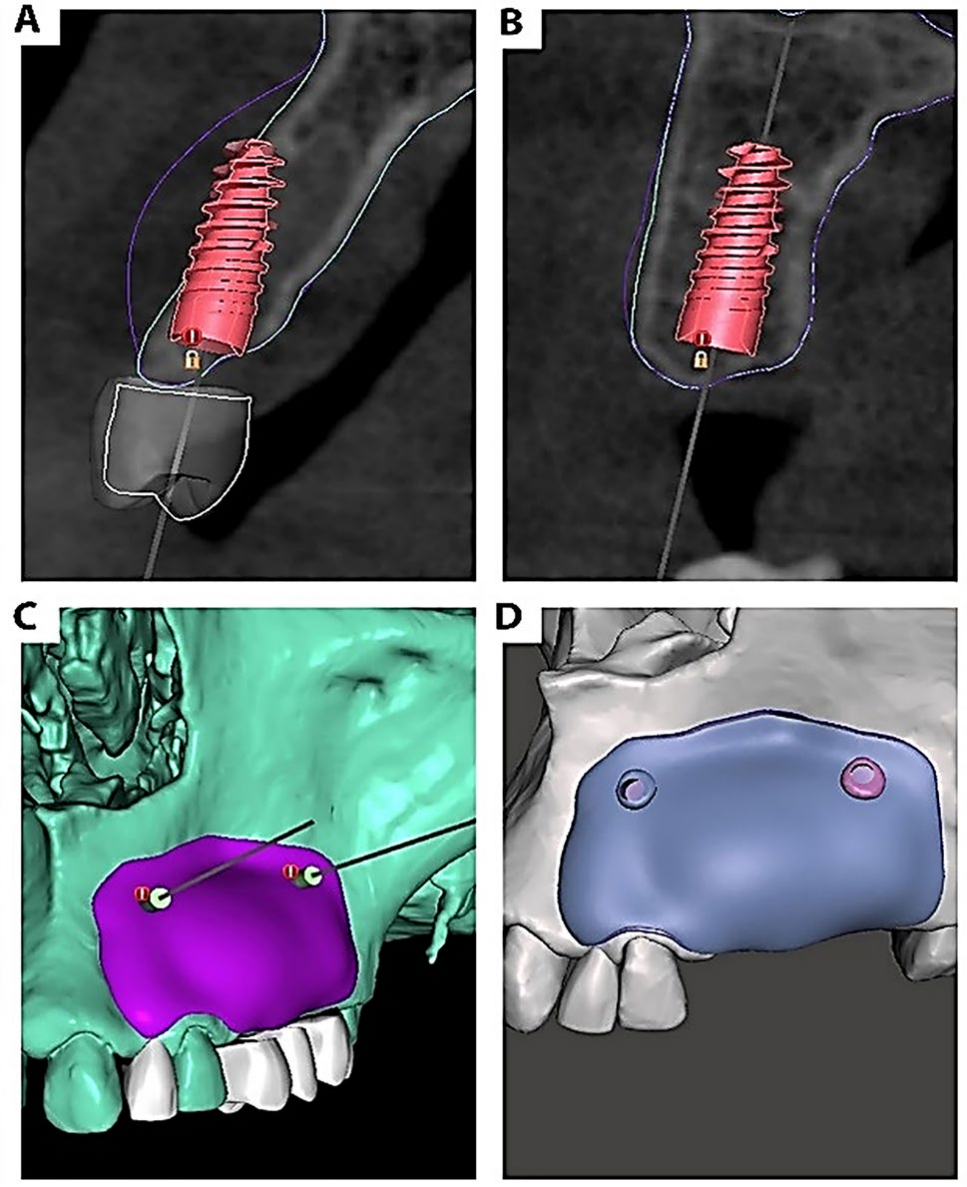
(**a**) & (**b**) Screenshots from Blue Sky Plan showing the superimposition of the virtually augmented model (blue outline) over the preoperative radiograph confirming the proper amount of augmentation over the implants (the distal implant (**b**) is used in this case only as a second reference for linear measurement analysis, as it didn’t need augmentation), (**c**) a screenshot from Blue Sky Plan showing the planning of the fixation screw positions, (**d**) a screenshot of Autodesk Meshmixer showing the final design of the membrane over the augmented mode

Afterward, the STL files were imported to a second open-source software, Autodesk Meshmixer^®^ (Meshmixer, Autodesk Inc., US), and each component of the file (implants, crowns, and bony model) was then separated as objects inside the same project. Using the (Draw) tool, the alveolar defect area was virtually augmented to the desired dimension to ensure the presence of sufficient bone around the virtually placed prosthetic-driven implant. The virtually augmented model was then exported as a new STL file. (Fig. 1d) For further verification, the virtually augmented model was again imported into the Blue-Sky Plan to ensure that the dimensions of the augmented model were larger than the diameter of the virtually placed implant. (Figure 2a and b)

Later, the area covered by the zirconia membrane was selected, and a 0.5 mm offset was used to create the fitting surface of the membrane. This offset layer was then separated, and a new connected offset was added to create the outer surface. After smoothening, the membrane thickness was decreased to 0.5 mm, which was then exported as a separate STL file. For a second verification, the new membrane STL file was imported again to the Blue Sky Plan^®^ to confirm the orientation of the membrane over the preoperative model and virtually placed on the implant. In addition, the fixation screws were virtually placed at the proper position away from the teeth roots or any vital structures and exported to an STL file. (Fig. 2c)

Finally, with the use of the Meshmixer^®^, the screw models were imported and automatically appended to the membrane. The screws were subsequently subtracted from the membrane via the (Boolean Difference) tool from the (Edit) menu, leading to the creation of screw holes. (Fig. 2d). The final design of the membrane was exported as a separate file and ready for milling. For evaluation procedures, a copy of the final membrane was combined with a copy of the virtually augmented model (CAD model) and exported as a separate STL file.

### Milling and preparation of the membrane

The final design of each membrane was exported to a CAD-CAM milling machine (Roland DWX 50^®^, Japan) to be milled and then inserted into a sintering oven (TABEO^®^-1/M/ZIRKON/100, MIHM-VOGT GmbH & Co. KG, Germany) for a 3-hour cycle. The moulding blanks used for milling of all membranes were Yttria Multi-Layered KATANA™ Zirconia (Kuraray, Miyoshi-cho, Miyoshi, Japan). The milling parameters were set as follows: spindle speed of 18,000 RPM, feed rate of 100 mm/min, and depth of cut of 0.1 mm per pass, using a 1.0-mm diamond-coated ball-end mill. A water-based coolant was applied continuously to minimize thermal stress and prevent micro-cracks in the pre-sintered zirconia.

After milling, zirconia membranes were finished and polished. The finishing process involved the use of a low-speed electric handpiece at 10,000 RPM with a series of diamond-coated rotary instruments (ZR Diamond, Komet Dental) in a sequence of coarse (100 μm grit) to fine (30 μm grit) burs under water cooling to remove surface irregularities and achieve a uniform thickness. Subsequently, polishing was performed using a two-step protocol with silicone-based polishing points (Diamond Ceramic Polishing Kit − 4540 A, Komet Dental) and a diamond polishing paste (Zirkopol, Ivoclar Vivadent) applied with a soft-bristled brush at 5000 RPM.

Before surgical procedures, the zirconia membranes were prepared as follows: rinsed, brushed under flowing water, immersed in pure disinfectant solution, rinsed, packaged in a single-use sterilization pack, and finally sterilized under a fractionated prevacuum at 134 °C for 18 min, with a drying time of 15 min.

### Surgical phase

**Fig. 3:**
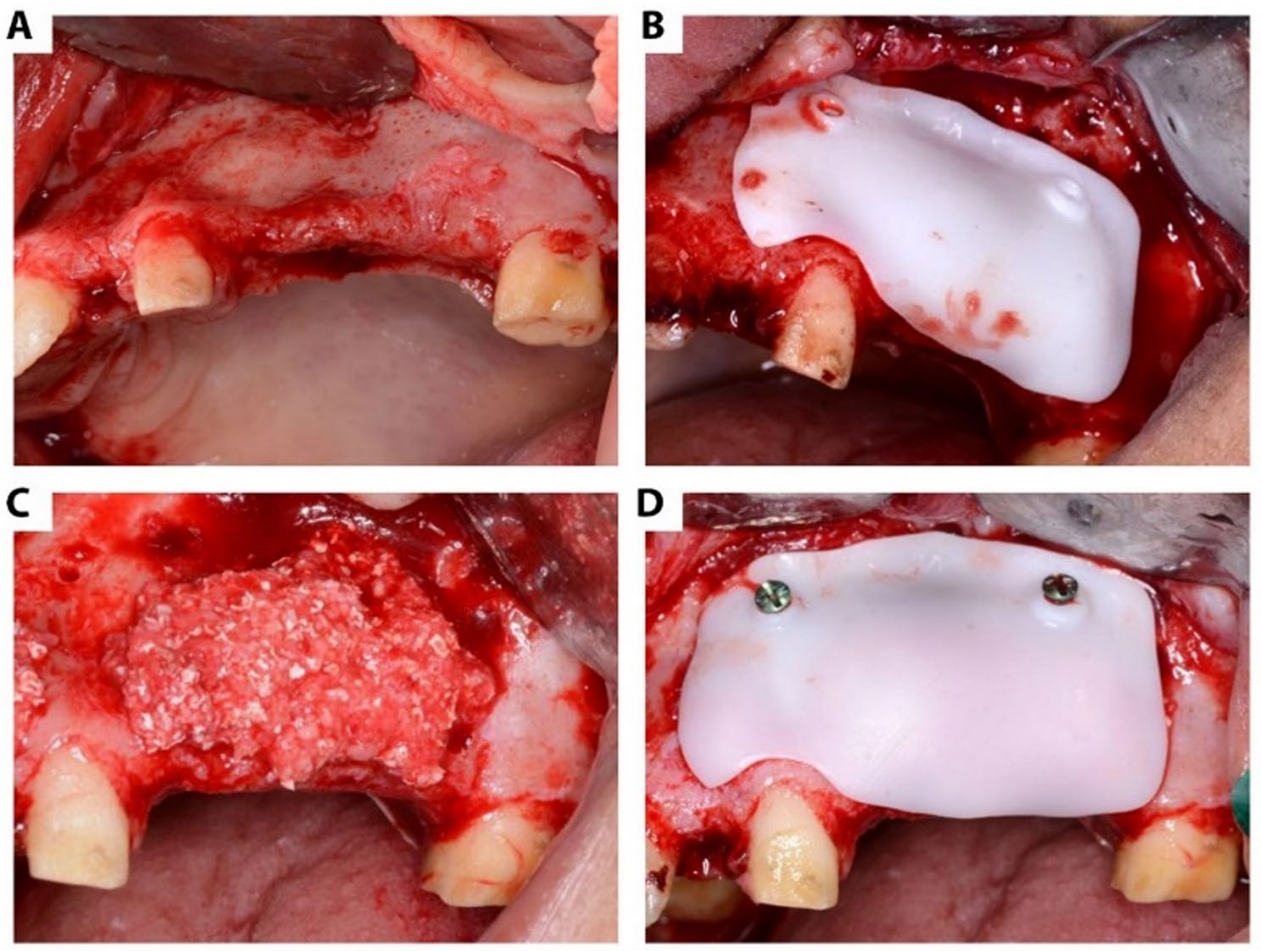
(**a**) Intraoperative photograph showing the alveolar defect (thin alveolar ridge), (**b**) intraoperative photograph showing the initial try-in of the zirconia membrane, (**c**) intraoperative photograph showing the placement of the graft particulate mixture, and (**d**) intraoperative photograph showing the zirconia membrane fixed in place

The procedures were performed under the effect of local anaesthesia with Articaine and 1:100000 epinephrine (Artpharmadent^®^, Artpharma Co., Egypt). A full thickness mucoperiosteal flap was reflected with one para crestal incision and two releasing incisions. (Fig. 3a) The initial try-in of the membrane was performed to ensure passive fitting of the membrane to the recipient site (Fig. 3b).

#### Harvesting the autogenous graft

Exposure of the external oblique ridge was performed through the elevation of a full thickness mucoperiosteal flap, and then, particles of the autogenous bone graft were harvested from the external oblique ridge via an Autochip Maker (ACM) (Neobiotech, USA, Inc.) followed by flap closure.

A small diameter round diamond bur mounted on a surgical handpiece was used to decorticate the alveolar bone of the defect area to provide a fresh and abundant blood supply to the following graft. The autogenous graft particles were mixed with xenograft particles (OneXeno^®^Graft Bovine, OneGraft^®^, Germany) at a mixing ratio of 1:1, and the mixture was subsequently divided into two parts. The first part was placed in layers at the deficient site, whereas the other part was placed inside the fitting surface of the membrane. The objective of this was to use the membrane as a template to ensure graft coverage of the whole alveolar defect. (Fig. 3c)

Self-taping micro fixation screws were used for membrane fixation, followed by removal of the excess of the graft mixture particles around the membrane peripheries. (Fig. 3d) Tension-free primary wound closure was achieved by minimal flap scoring. Tension-free primary closure over the membrane was performed via 4/0 vicryl interrupted sutures (EGYSORB^®^, TAISER-MED Co. Egypt).

- **Postoperative care**:

For all patients, a standard regimen of treatment, the antibiotic Amoxicillin 857 mg Clavulanate 125 mg (Augmentin^®^, GlaxoSmithKline, UK.) A total of 1 g twice daily and nonsteroidal anti-inflammatory Ibuprofen 600 mg (Brufen^®^, Abbott, Inc.) twice daily for at least five days and Chlorohexidine mouthwash (Orovex-H ^®^ Mouth Wash, MACRO Pharmaceuticals, Cairo, Egypt), starting the day after surgery, for seven days were prescribed for all patients. Cold fomentations were applied on the first day of the operation over the skin covering the surgical field every 20 min. The patient should be instructed strictly to avoid any physical activities or sports exercises that could cause external trauma, falls or similar impacts but encouraged for early mobilization and walking. All patients were instructed to maintain good oral hygiene and return to remove the sutures after ten days. Patients were informed to have a follow up visit every month along a period of 6 months before the second surgery that included the membrane removal and implant placement.

### Preoperative (workflow parameters)

All data concerning the preoperative workflow for custom membrane preparation, such as the design time in hours, milling time in hours, and total time after adding the sintering and sterilization times, were recorded.

### Evaluation of membranes accuracy

All patients were asked to have immediate (1 week) postoperative CBCT. The membrane accuracy was evaluated by intraoperative clinical evaluation and by postoperative (CBCT).

### Clinical evaluation

All the membranes were checked intraoperatively to confirm their accuracy and proper passive fit to the targeted area without interference from neighbouring teeth, bone, or both.

### Radiographic evaluation

#### Volumetric analysis

A segmented immediate postoperative bony model from the immediate postoperative CBCT image was exported via the Blue Sky Plan^®^. Volumetric analysis was performed with an Autodesk Meshmixer^®^ via the (Stability) function, which automatically measures the volume of the virtually augmented area after its separation from the CAD-augmented model and the immediate postoperative augmented area after its separation from the immediate postoperative model.

#### Linear radiographic measurements

**Fig. 4:**
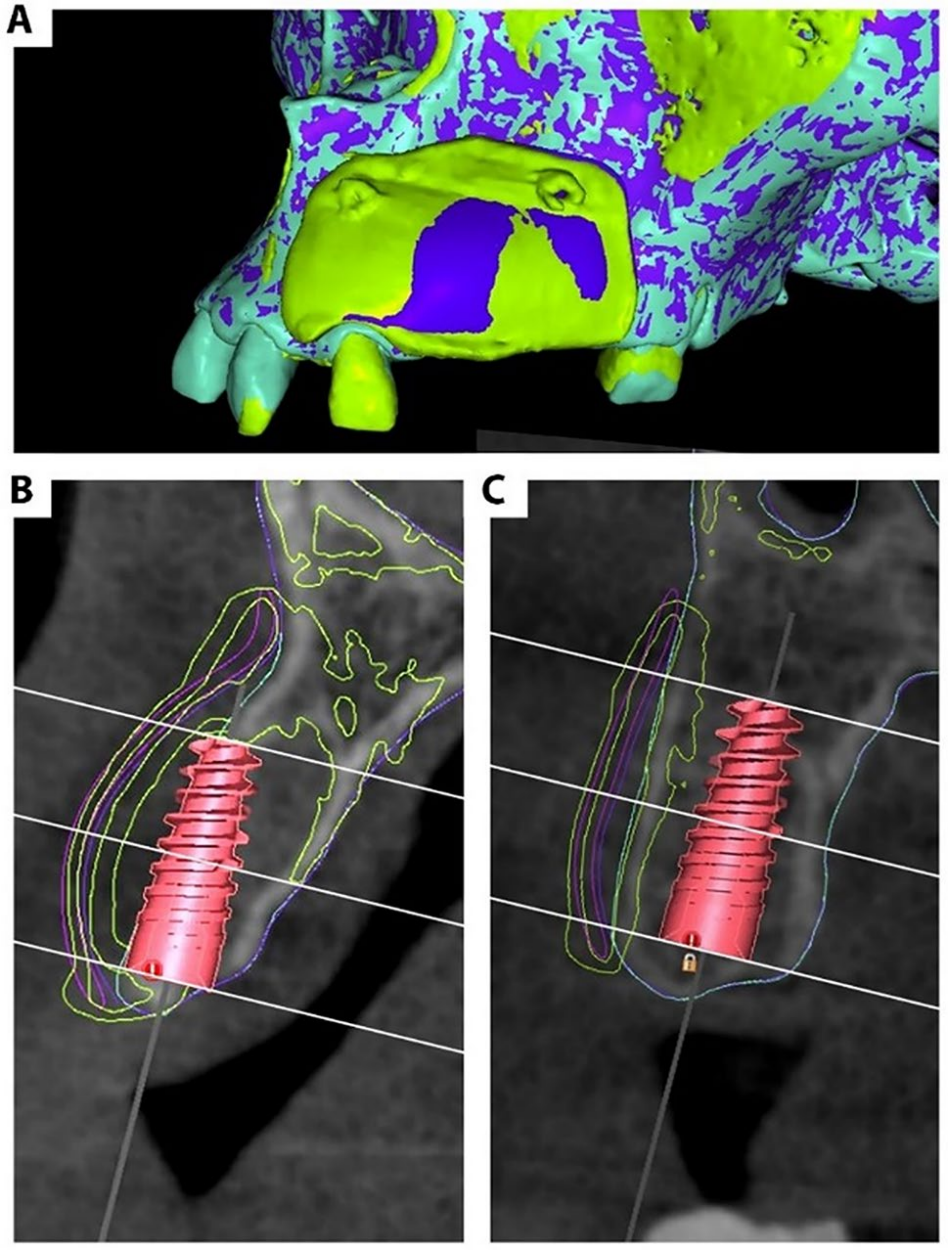
(**a**) A screenshot from Blue Sky Plan showing the 3D view of the superimposition of the preoperative model, virtually augmented model and virtual membrane (blue) and the immediate postoperative model (green), (**b**) & (**c**) screenshots from Blue Sky Plan showing the tangential cross section of both virtual implants in places and their relation to the superimposed virtual augmented model and virtual membrane (blue outline) and the immediate postoperative model (green outline). The three horizontal lines correspond to the three levels where horizontal measurements were recorded (from the midline of the implant to the outer border of both membranes). The vertical line at the middle of the implant where the vertical measurements were recorded

Both virtual preoperative and actual immediate postoperative models were imported into Blue Sky Plan^®^ and then superimposed on each other in the preoperative CBCT for linear horizontal and vertical measurements to detect the difference between the position of the planned membrane and the actual position of the surgically placed membrane. The reference point of these measurements was the preoperative virtually placed implant using the central line passing through the implant screw channel in the tangential cross-sectional view and three horizontal lines: at the implant platform, at the middle of the implant length, and at the apex of the implant. The vertical measurement was performed at the central line of the virtual implant that passed through the implant screw channel. Horizontal and vertical measurements were recorded from the virtually placed implant to the outer border of both membranes. In patients who were planned to have more than one implant, the measurements were recorded regarding each virtually placed implant, and the mean measurements were used for the patient. (Fig. 4)

In immediate postoperative CBCT, the CAD model was imported and registered to the immediate postoperative radiograph to check the actual position of the fixation screws in relation to their holes in the virtual membrane.

### Statistical analysis

All the data obtained from the volumetric and linear measurements were statistically analysed and presented in the form of tables via the IBM Statistical Package for Social Science (SPSS) software version 23 (Armonk, NY: IBM Corp.). Paired t-test was used to compare volume, horizontal and vertical measurements. Means and standard deviations were used in the analysis of demographic data and perioperative workflow parameters.

## Results

Twelve patients, six females and six males, underwent computer-guided maxillary alveolar ridge augmentation. Two implants were planned for four patients, three implants were planned for one patient, and a single implant was planned for seven patients (a total of 18 implants were planned). Eight patients had augmentation in the upper anterior region, and four patients had augmentation in the premolar‒molar region. The age range was from 25 to 46 years, with a mean age of 32.42 ± 6.02 years. (Table 1)

**Table 1:** Demographic data of the patients and implant distribution

Patient	Gender	Age (years)	site	Number of implants
#1	Male	35	Upper anterior region	1
#2	Male	33	Upper anterior region	1
#3	Male	26	Upper anterior region	2
#4	Female	29	Upper anterior region	1
#5	Female	27	Upper anterior region	1
#6	Female	30	Upper premolar/molar region	2
#7	Female	45	Upper premolar/molar region	3
#8	Male	33	Upper anterior region	2
#9	Female	40	Upper premolar molar region	2
#10	Male	25	Upper anterior region	1
#11	Male	29	Upper premolar region	1
#12	Female	37	Upper anterior region	1
Mean ± SD		**32.42 ± 6.02**		

### Clinical intraoperative evaluation

All twelve membranes had excellent passive fit to the alveolar defects without any interference with the neighbouring teeth and/or surrounding bony structures and had virtually planned orientations without interference or need for modification. All twelve surgeries were performed without intraoperative and/or postoperative complications (excessive bleeding, infection, injury to neighbouring vital structures, or wound dehiscence).

### Radiographic volumetric measurements

A comparison between the virtually augmented model volume and the immediate postoperative augmented model volume revealed no statistically significant difference (*P* = 0.628), where the P value was considered significant at < 0.05 (Table 2).

### Radiographic linear measurements

The actual custom membrane was placed on the immediate postoperative model either in the horizontal or vertical direction. All screws in the immediate postoperative model (real screws) passed through their planned screw holes in the designed virtual membrane. This finding was proved statistically as there was no statistically significant difference either in the position or the orientation between the virtually planned membrane in place on the CAD-augmented model and the actual custom membrane in the horizontal and vertical directions. The p values were 0.226 and 0.239 horizontally and vertically, respectively. P values < 0.05 were considered significant (Table 2).

**Table 2:** Comparison between the virtually planned and actually milled and used zirconia membrane measurements (volume, horizontal, and vertical measurements)

Parameter	Virtually planned membrane (*N* = 12)	Actually used membrane intraoperatively (*N* = 12)	(*P*) Value
Mean augmented models Volume ± SD	935.917 ± 382.752 mm^3^	962.333 ± 411.265 mm^3^	0.628
Mean horizontal measurements ± SD	5.221 ± 0.729 mm	5.439 ± 0.905 mm	0.226
Mean Vertical Measurements ± SD	2.754 ± 0.764 mm	3.01 ± 0.691 mm	0.239

Paired student t-test, SD = standard deviation, P = significance when < 0.05, 95% confidence interval

mm = millimeter

### Preoperative workflow parameters

The designing time was reduced from 4 h for the first case to 22 min for the final case, with a (mean ± standard deviation) time of 1 h and 19 min ± 1 h and 12 min. The maximum milling time was 53 min, and the minimum was 22 min, with a (mean ± standard deviation) time of 28 ± 11 min. The sintering time and sterilization time were fixed at 3 h, and 33 min, respectively, for all the membranes.

The maximum total time of the preoperative steps (design, milling, sintering and sterilization) was 8.13 h, whereas the minimum time was 4 h and 10 min, with a (mean ± standard deviation) time of 5 h and 20 min ± 1 h and 15 min. (Table 3).

**Table 3:** Preoperative workflow parameters (designing time, milling time, and total time)

Patient	Designing time in hours (hrs)	Milling Time in hours (hrs)	Total time in hours after adding (sintering and sterilization time)
#1	4	0.58	8.13
*#2*	3	0.52	7.07
*#3*	2.5	0.41	6.46
*#4*	1.5	0.32	5.37
*#5*	1	0.35	4.91
*#6*	0.83	0.75	5.13
*#7*	0.75	0.37	4.67
*#8*	0.5	0.45	4.56
*#9*	0.5	0.87	4.92
*#10*	0.35	0.33	4.18
#11	0.5	0.34	4.34
#12	0.33	0.35	4.18
Mean ± SD	1.313 ± 1.207	0.472 ± 0.180	5.331 ± 1.246

SD = standard deviation

## Discussion

Computer-guided GBR has been applied to design and fabricate diverse types of rigid membranes. One such material is zirconia membranes, which exhibit outstanding biological and mechanical properties in addition to high affinity for fibroblasts [13]. Many previous studies [10, 12, 13] have used zirconia membranes designed with different software; most of them have a high cost of purchase. Owing to the widespread use of 5-axis milling machines in dental laboratories and the relatively lower cost and availability of zirconia, producing accurately milled patient-specific membranes using this material is a more convenient technique than producing custom-made titanium meshes [17]. The use of a mixture of autogenous and xenograft materials as the grafting material was mentioned in multiple previous studies concerning GBR with either resorbable or nonresorbable barriers, with highly predictable results [18–21]. 

This study provides a free-of-charge digital workflow for designing and fabrication of zirconia membranes and evaluating the design accuracy by comparing the immediate postoperative position and orientation of the surgically placed membrane to the virtually placed membrane in the preoperative phase. The authors used two free open-source software programs, Blue Sky Plan^®^, for implant planning, model segmentation, verification of membrane design and placement and recording the differences in horizontal and linear measurements for both membranes. The other program is Autodesk Meshmixer^®^ for model virtual augmentation, design of membranes and volumetric analysis of both virtually and actually augmented volume.

The integration between these two software programs coincides with previous studies that used CAD-designed membranes either fabricated from zirconia membranes or other materials [10, 12–15, 22]. These studies used the integration of multiple software one for (DICOM) file viewing, model segmentation and exporting of STL files, whereas other software(s) were used for model augmentation and membrane design. However, this study is distinguished by introducing a totally free digital workflow.

This study tested an effective methodology for early accuracy evaluation of zirconia membrane placement at the immediate postoperative stage rather than waiting from 6 to 9 months for bone formation to evaluate the accuracy of the digital workflow and surgical procedures, as performed in previous studies, either to investigate the use of zirconia membranes or any other CAD membrane [10, 13]. This was achieved via volumetric analysis of both the virtually augmented model and the immediate postoperative model. Additionally, virtually placed implants were used in preoperative CBCT as stable reference points for accurate measurements in the two stages of evaluation (virtual preoperative augmentation and immediate postoperative), making good use of the ability of model registration. Another precise reference introduced in this study was the actual position of the fixation screws in relation to the planned screw holes in the virtually designed membrane.

The thickness of the zirconia membrane was consistent in all cases, ranging from 0.4 to 0.5 mm, as demonstrated by Mandelli et al. [13]. The mesio-distal and labio-palatal dimensions varied between cases on the basis of the three-dimensional configuration of the bony defect and the amount of bone augmentation needed to achieve appropriate three-dimensional implant placement in the future.

The intraoperative evaluation of all the membranes revealed excellent exact passive fitting to the targeted positions without interference with any neighbouring anatomical structure and/or neighbouring teeth, facilitating the surgical procedure.

This study supports the use of radiographic volumetric analysis of the augmented bone as a main guide to confirm the accuracy of the membrane design. The significant difference in the volume of the immediate postoperative augmented volume indicates the defect in the design process and the accuracy of both software programs. Since there is no statistically significant difference between the immediate postoperative augmented bone volume and the virtually augmented volume (*P* = 0.628), the accuracy of both software programs, the design process and the surgical procedure can be confirmed.

The linear measurements revealed that the statistical analysis of the deviations between the planned CAD virtual membrane and the actual membrane yielded no significant difference in either the horizontal or vertical dimensions (*P* = 0.226 and 0.239, respectively). Another evidence of the accuracy of the design is that all the actual screws were confirmed to pass through their planned openings in the CAD virtual membranes. This finding could not be achieved unless the actual membrane was in the designed position and orientation, which added another clinical value in that all screws were confirmed to be positioned away from any neighbouring vital structure as planned in the preoperative phase, which ensured intra- or postoperative complications.

The time required for membrane design and surgeon training are crucial factors. For our first cases, it took approximately 3–4 h per case, whereas the design time in the last cases was approximately 20 min. Given that the total time required for the preoperative workflow steps for the first case, including membrane design, milling, sintering and sterilization, was 8 h and 8 min, and that for the last case, was 4 h and 11 min, the results of this study support that the whole process, including the surgical phase, can be performed on the same day, which adds important clinical value, where the (mean ± standard deviation) total time after adding the fixed sintering and sterilization time for all cases was 5 h and 20 min ± 1 h and 15 min.

It is important to consider the costs associated with the required data collection for the design. However, the only data used were CBCT, which is routinely used during the planning of implant placement for any case. Additionally, the two software programs used for the design were free of charge and could be easily obtained through the official websites of the applications.

Despite the promising results of this study, there are limitations, such as the need for more research with larger sample sizes, the use of other anatomical positions, including mandibular defects, and the use of this technique for testing the accuracy of the design of other nonrigid membranes, such as CAD titanium meshes or PEEK.

## Conclusion

This study was conducted to evaluate the accuracy of a technique that enables operators to design a zirconia membrane for computer-guided maxillary GBR by using two free open-source software programs. Furthermore, the postoperative outcome was validated by comparing the position and orientation of the actual membrane with the preoperatively designed virtual model, confirming that the achieved results matched the planned objectives. Future research should be directed toward the use of the open-source digital workflow with zirconia membranes in other anatomical alveolar defects including mandibular defects with larger sample size and more long term follow-up pf the GBR process, the used digital workflow should be investigated to design and evaluate other custom-made rigid GBR membrane materials such as PEEK and custom-made titanium meshes, finally the accuracy of the used software programs should be investigated in comparison with other software programs.

## Electronic supplementary material

Below is the link to the electronic supplementary material.

Supplementary Material 1

## Data Availability

All data generated or analysed during this study are included in this published article [and its supplementary information files].

## Declarations

### Adherence to CONSORT guidelines

The authors state that the study adheres to CONSORT guidelines for reporting clinical trials.

### URLs for downloading the software programs of the study

*Blue Sky Plan*:

https://blueskybio.com/pages/free-treatement-planning-software?srsltid=AfmBOopolItZBeUNC-z1bpx2xofU7dgyAua9ZjWWF-ZqJG6iIA7b3VxM.

Autodesk Meshmixer:

https://apps.autodesk.com/FUSION/en/Detail/Index?id=410892018526193510026appLang=en26os=Win64.

## Abbreviations

GBR: Guided Bone Regeneration
CBCT: Cone Beam Computed Tomography
CAD: Computer-Aided Design
CAD/CAM: Computer-Aided Design/Computer-Aided Manufacturing
STL: Standard Tessellation Language
DICOM: Digital Imaging and Communications in Medicine
e-PTFE: expanded Polytetrafluoroethylene
d-PTFE: High-Density Polytetrafluoroethylene
PEEK: Poly-Ether-Ether-Ketone
SPSS: Statistical Package for Social Science
kV: kilo-volt
mA: milliampere
LP/mm: Line Pairs per Millimeter
MTF: Modulation Transfer Function
RPM: Revolutions Per Minute
µm: Micrometer (Micron)
SD: Standard Deviation
mg: Milligram
h: Hours
min: Minutes

## References

[1] Retzepi M, Donos N. Guided bone regeneration: biological principle and therapeutic applications. Clin Oral Implants Res. 2010;21(6):567–76. 20666785 10.1111/j.1600-0501.2010.01922.x

[2] Rakhmatia YD, Ayukawa Y, Furuhashi A, Koyano K. Current barrier membranes: titanium mesh and other membranes for guided bone regeneration in dental applications. J Prosthodont Res. 2013;57(1):3–14. 23347794 10.1016/j.jpor.2012.12.001

[3] Briguglio F, Falcomatà D, Marconcini S, Fiorillo L, Briguglio R. Farronato. The use of titanium mesh in guided bone regeneration: a systematic review. Int J Dent. 2019;2019:9065423. 30881455 10.1155/2019/9065423 PMC6383423

[4] von Arx T, Kurt B. Implant placement and simultaneous ridge augmentation using autogenous bone and a micro titanium mesh: a prospective clinical study with 20 implants. Clin Oral Implants Res. 1999;10(1):24–33. 10196787 10.1034/j.1600-0501.1999.100104.x

[5] Louis PJ, Gutta R, Said-Al-Naief N, Bartolucci AA. Reconstruction of the maxilla and mandible with particulate bone graft and titanium mesh for implant placement. J Oral Maxillofac Surg. 2008;66(2):235–45. 18201602 10.1016/j.joms.2007.08.022

[6] Jain S, Choudhary K, Nagi R, Shukla S, Kaur N, Grover D. New evolution of cone-beam computed tomography in dentistry: combining digital technologies. Imaging Sci Dent. 2019;49(3):179–90. 31583200 10.5624/isd.2019.49.3.179 PMC6761063

[7] Sheikhi M, Karami M, Abbasi S, Moaddabi A, Soltani P. Applicability of cone beam computed tomography Gray values for Estimation of primary stability of dental implants. Braz Dent Sci. 2021;24(1):8. P- P.

[8] Kim M-J, Jeong JY, Ryu J, Jung S, Park H-J, Oh H-K, et al. Accuracy of digital surgical guides for dental implants. Maxillofac Plast Reconstr Surg. 2022;44(1):35. 36282400 10.1186/s40902-022-00364-4 PMC9596667

[9] Bai X, Wu T, Zhu Y, Yang C, Cheng T, Liu Y, et al. Cone-wedge anchored surgical templates for stackable metal guide: a novel technique. Int J Implant Dent. 2024;10(1):27. 38819712 10.1186/s40729-024-00539-w PMC11143131

[10] Malmström J, Anderud J, Abrahamsson P, Wälivaara DÅ, Isaksson SG, Adolfsson E. Guided bone regeneration using individualized ceramic sheets. Int J Oral Maxillofac Surg. 2016;45(10):1246–52. 27364369 10.1016/j.ijom.2016.06.005

[11] Cionca N, Hashim D, Mombelli A. Zirconia dental implants: where are we now, and where are we heading? Periodontol 2000. 2017;73(1):241–58. 28000266 10.1111/prd.12180

[12] Melek LN. Comparison of two pre-prosthetic surgical techniques for augmentation of mandibular vertical ridge defects. JJoO. 2022;14(1):59–63.

[13] Mandelli F, Traini T, Ghensi P. Customized-3D zirconia barriers for guided bone regeneration (GBR): clinical and histological findings from a proof-of-concept case series. J Dent. 2021;114:103780. 34400253 10.1016/j.jdent.2021.103780

[14] Li L, Wang C, Li X, Fu G, Chen D, Huang Y. Research on the dimensional accuracy of customized bone augmentation combined with 3D-printing individualized titanium mesh: A retrospective case series study. Clin Implant Dent Relat Res. 2021;23(1):5–18. 33336492 10.1111/cid.12966

[15] Mounir M, Shalash M, Mounir S, Nassar Y, El Khatib O. Assessment of three dimensional bone augmentation of severely atrophied maxillary alveolar ridges using prebent titanium mesh vs customized poly-ether‐ether‐ketone (PEEK) mesh: A randomized clinical trial. Clin Implant Dent Relat Res. 2019;21(5):960–7. 30895678 10.1111/cid.12748

[16] Felice P, Lizio G, Barausse C, Roccoli L, Bonifazi L, Pistilli R, et al. Reverse guided bone regeneration (R-GBR) digital workflow for atrophic jaws rehabilitation. Appl Sci. 2022;12(19):9947.

[17] Hofferber CE, Beck JC, Liacouras PC, Wessel JR, Getka TP. Volumetric changes in edentulous alveolar ridge sites utilizing guided bone regeneration and a custom titanium ridge augmentation matrix (CTRAM): a case series study. Int J Implant Dent. 2020;6:1–9. 33300105 10.1186/s40729-020-00269-9 PMC7726098

[18] Pandit N, Pandit IK. Autogenous bone grafts in periodontal practice: A literature review. J Int Clin Dent Res Org. 2016;8(1):27–33.

[19] Kim Y-K, Ku J-K. Guided bone regeneration. J Korean Assoc Oral Maxillofac Surg. 2020;46(5):361–6. 33122463 10.5125/jkaoms.2020.46.5.361 PMC7609932

[20] Kim Y-K, Kim S-G, Lim S-C. The comparative study of guided bone regeneration using various of bone graft materials. J Korean Assoc Oral Maxillofac Surg. 2007:350–8.

[21] Mordenfeld A, Johansson CB, Albrektsson T, Hallman M. A randomized and controlled clinical trial of two different compositions of deproteinized bovine bone and autogenous bone used for lateral ridge augmentation. Clin Oral Implants Res. 2014;25(3):310–20. 23551390 10.1111/clr.12143

[22] El Morsy OA, Barakat A, Mekhemer S, Mounir M. Assessment of 3-dimensional bone augmentation of severely atrophied maxillary alveolar ridges using patient‐specific Poly ether‐ether ketone (PEEK) sheets. Clin Implant Dent Relat Res. 2020;22(2):148–55. 32103625 10.1111/cid.12890

